# Model-guided development of pharmacokinetic/pharmacodynamic cut-offs and evaluation of sitafloxacin dosing regimens against target pathogens

**DOI:** 10.3389/fphar.2025.1476158

**Published:** 2025-02-14

**Authors:** Hailan Wu, Yi Li, Xin Li, Yaxin Fan, Beining Guo, Xiaofen Liu, Wanzhen Li, Mengting Chen, Yan Chen, Jing Zhang

**Affiliations:** ^1^ Institute of Antibiotics, Huashan Hospital, Fudan University, Shanghai, China; ^2^ Key Laboratory of Clinical Pharmacology of Antibiotics, National Health Commission of the People’s Republic of China, Shanghai, China; ^3^ National Clinical Research Center for Geriatric Diseases, Huashan Hospital, Fudan University, Shanghai, China; ^4^ Phase I Clinical Research Center, Huashan Hospital, Fudan University, Shanghai, China

**Keywords:** sitafloxacin, PK/PD cut-offs, population pharmacokinetics, monte carlo simulation, infected patients, probability of target attainment, cumulative fraction of response

## Abstract

**Introduction:**

The establishment of clinical breakpoints for antimicrobial drug is crucial for guiding appropriate therapeutic interventions. This study aims to identify the pharmacokinetic/pharmacodynamic (PK/PD) cut-offs for using sitafloxacin against target pathogens to support clinical breakpoint establishment for antimicrobial drug sensitivity testing.

**Methods:**

A population PK (PopPK) model was built (342 subjects) to calculate the dosing-regimen-dependent (50 mg q12 h, 100 mg q24 h and 100 mg q12 h) PK parameters of sitafloxacin-infected patients, which were combined with *in vitro* PD data and PK/PD target data. The probabilities of attainment (PTAs) and cumulative fraction of response (CFR) values for different sitafloxacin dosing regimens against *Streptococcus pneumoniae, Staphylococcus aureus, Escherichia coli, Klebsiella pneumoniae* and *Pseudomonas aeruginosa* were calculated via Monte Carlo simulation.

**Results:**

PopPK modelling revealed that the PK profile of sitafloxacin was consistent with a two-compartment model with first-order elimination. Creatinine clearance affected total clearance, bodyweight and age affected the central ventricular apparent volume of distribution, and food affected the sitafloxacin absorption rate. On the basis of the animal infection model target (*f*AUC_24h_/MIC = 11.56), the anti-*Streptococcus pneumoniae* sitafloxacin dosing regimen PTAs were >95% (MIC ≤ 0.06, ≤0.06, ≤0.125 mg/L; CFRs = 98.2∼99.3%). With a clinical study target of *f*AUC_24h_/MIC ≥ 30, the anti-*Streptococcus pneumoniae* dosing regimen PTAs were >95% (MIC ≤ 0.03, ≤0.03, ≤0.06 mg/L; CFRs = 89.2∼97.3%). For the other four strains, the dosing-regimen-dependent sitafloxacin PK/PD cut-offs were 0.06, 0.06 and 0.125 mg/L, respectively (CFRs = 56.3∼76.9%).

**Discussion:**

Our findings suggest that sitafloxacin PK/PD cut-offs of S ≤ 0.06 mg/L and R > 0.125 mg/L should be used against these five strains and that the sitafloxacin dosing regimens (50 mg q12 h, 100 mg q24 h and 100 mg q12 h) have the expected efficacy against *Streptococcus pneumoniae*-related infections, but the efficacy against *Pseudomonas aeruginosa*-associated infections needs to be verified in clinical practice.

## 1 Introduction

Antimicrobial susceptibility clinical breakpoints are among the most important components of antimicrobial precision therapy. The qualitative reporting of a microbe as sensitive (S), moderately sensitive (I), or resistant (R) to an antimicrobial agent provides an important basis for clinicians to select antimicrobial drugs for the treatment of pathogenic bacterial infections. The European Committee on AST (EUCAST) and Clinical and Laboratory Standards Institute (CLSI) have proposed that the clinical breakpoint should be determined by balancing the epidemiological cut-off (ECOFF), pharmacokinetic/pharmacodynamic cut-off (PK/PD_CO_) and clinical cut-off (Turnidge and Paterson, 2007). The ECOFF, also known as the wild-type cut-off (CO_WT_), is usually the upper limit of the minimum inhibitory concentration (MIC) of a wild-type population, and it can be used to differentiate wild-type strains from non-wild-type strains. On the basis of the PK/PD index and targets that are most relevant to clinical efficacy, the PK/PD cut-off can be estimated as the highest MIC value for a dosing regimen with a high probability of target attainment (PTA). Currently, the acceptable PTA is still under debate, and PTAs of 99%, 95% or 90% have all been used (Asín et al., 2012; Frei et al., 2008; Mouton et al., 2012). Moreover, a clinical cut-off is determined by an MIC value related to the clinical therapeutic outcomes of clinical trials. The breakpoint-setting organization committee integrates these data to make the final decision on clinical breakpoints.

Sitafloxacin, a fourth-generation fluoroquinolone antibacterial drug, is marketed in Japan, Thailand and China for the treatment of pneumonia and secondary infections causing chronic respiratory diseases and is used at a dosage of 50 mg q12 h or 100 mg q24 h or q12 h. Sitafloxacin has a broad-spectrum antibacterial effect, with antimicrobial activity against aerobic and anaerobic gram-positive and gram-negative bacteria, such as *Mycoplasma pneumoniae* and *Chlamydia pneumoniae* (Li et al., 2022a; Li et al., 2022b). It works by inhibiting DNA gyrase and topoisomerase IV activity and is superior to other quinolones in terms of inhibitory activity (Kanda et al., 2008). Sitafloxacin PKs are characterized by rapid absorption and wide distribution, with approximately 70% of the drug remaining in its original form upon excretion in urine. To our knowledge, no study has established accurate clinical breakpoints for sitafloxacin against Gram-positive and gram-negative bacteria following the guidelines of the EUCAST and CLSI. One study reported sitafloxacin susceptibility trends in a nationwide collection of clinical isolates from 1994 to 2016 in Japan; however, the lack of internationally recognized sitafloxacin susceptibility breakpoints makes targeted clinical therapy difficult. Therefore, establishing sitafloxacin susceptibility breakpoints is necessary to provide a reference for clinical treatment.

Currently, the ECOFFs for sitafloxacin against *Streptococcus pneumoniae, Staphylococcus aureus, Escherichia coli, Klebsiella pneumoniae* and *Pseudomonas aeruginosa* are 0.125, 0.125, 0.03, 0.06 and 0.5 mg/L, respectively, which have been published on the official EUCAST website (https://mic.eucast.org/search/). The clinical cut-off for sitafloxacin was derived from prospective clinical studies comparing clinical and bacteriological prognoses for infections caused by pathogens with different MICs in infected Japanese and Chinese patients. The clinical and microbiological efficacy of sitafloxacin in the treatment of Japanese patients with community-acquired pneumonia is greater than 90%, with MIC ranges of ≤0.025–0.39, ≤0.025, ≤0.025–0.78, and 0.11–0.78 mg/L against clinically isolated *Streptococcus pneumoniae, Staphylococcus aureus, Klebsiella pneumoniae,* and *Pseudomonas aeruginosa* strains, respectively (Tanigawara et al., 2013). The clinical and microbiological efficacy of sitafloxacin in the treatment of Chinese patients with community-acquired pneumonia is >94%, with MIC_90_ values of 0.125, ≤0.06 and 0.25 mg/L against clinically isolated *Streptococcus pneumoniae, Staphylococcus aureus* and *Klebsiella pneumoniae* strains, respectively (Li et al., 2021). Sitafloxacin showed good clinical (>81.8%) and microbiological (>93.3%) efficacy in the treatment of Chinese patients with acute uncomplicated or complicated urinary tract infections, with an MIC_90_ of 1 mg/L against clinically isolated *Escherichia coli* (Li et al., 2020). However, the PK/PD cut-offs for sitafloxacin have not been studied.

Therefore, the aim of this study was to calculate the steady-state PK parameters of sitafloxacin in infected patients under different dosing regimens (50 mg q12 h, 100 mg q24 h, 100 mg q12 h) by establishing population PK (PopPK) models and to obtain PK/PD cut-off values for sitafloxacin against target pathogens via PK/PD analyses, which could provide a basis for determining clinical breakpoints for drug sensitivity and evaluating the current dosing regimen.

## 2 Results

### 2.1 Fundamental data

A total of 342 subjects were included in this PopPK dataset of sitafloxacin treatment outcomes, including 147 patients with respiratory system infection (Japan), 12 subjects with renal insufficiency (Japan), 135 healthy subjects from Japan, and 48 healthy subjects from China. A total of 3,294 plasma concentration data points were included in the PopPK dataset (the overall percentage of missing and excluded concentration data was 1.02%). Among the 342 subjects, 246 (71.9%) were male, and 96 (28.1%) were female. The demographic baseline values are presented in Table 1.

**TABLE 1:** Mean (SD) baseline demographic characteristics of the subjects included in the NONMEM dataset.

Characteristics	Mean ± SD
Age (years)	43.0 ± 21.7
Body weight (kg)	58.2 ± 10.2
Height (cm)	164.7 ± 9.6
BMI (kg/m^2^)	21.4 ± 3.0
CRCL (mL/min)	115.1 ± 53.5

Abbreviations: BMI, body mass index; CRCL, creatinine clearance rate; SD, standard deviation.

### 2.2 Population PK modelling

The PopPK model was constructed using a dataset composed of 3,294 plasma samples from 342 subjects. A two-compartment model with a linear elimination model best described sitafloxacin PKs after administration to the subjects. The absorption of sitafloxacin involves zero-order and first-order kinetics.

Statistically significant covariate effects were identified and retained in the final model: clearance (CL) on the creatinine clearance rate (CRCL) and age and weight (WT) on the apparent volume of the central compartment (V2). The zero-order absorption phase of drugs is influenced by food consumption status. The final PopPK parameters are summarized in Table 2, and the final sitafloxacin PopPK model is expressed as follows:

$$\text{CL}=14.7\times {\left(\frac{\text{CRCL}}{106.88}\right)}^{0.46}\times e^{\eta_{1}}\;\left(L/h\right)$$

$$V_{2}=89.8\times {\left(\frac{\text{WT}}{58.55}\right)}^{0.966}\times {\left(\frac{\text{AGE}}{31}\right)}^{0.286}\times e^{\eta_{2}}\;\left(L\right)$$

$$V_{3}=33.8\;\left(L\right)$$

$$Q=5.81\;\left(L/h\right)$$

$$D_{1}=0.281\times {\left(1+\text{FOOD}\right)}^{1.59}\times e^{\eta_{3}}\left(h\right)$$

$$\text{TLAG}=0.205\;\left(h\right)$$

$$\text{Absorption}=\left\{\begin{array}{l} \text{Zero}-\text{order absorption}:\text{in fasting state }D1\left(h\right)=0.281\\ \text{in fed state }D1\left(h\right)=0.846\\ \text{First}-\text{order absorption}:\text{Ka }\left(h^{-1}\right)=5.19 \end{array}\right.$$

Note: Ka, absorption rate constant; TLAG, lag time in absorption; V2, central volume of distribution; CL, clearance; Q, intercompartmental clearance; V3, peripheral volume of distribution; D1, zero-order absorption rate.

**TABLE 2:** Parameter estimates and bootstrap validation of the final PopPK model of sitafloxacin.

Parameter	Unit	Model estimates	Bootstrap validation		Deviation (%)
		Mean (RSE%)	Mean	95% CI	
PK parameters					
Ka	—	5.19 (16.3)	5.16	4.02–6.44	−0.6
TLAG	h	0.205 (1.1)	0.207	0.196–0.219	1.0
V2	L	89.8 (2.8)	89.6	84.2–95.6	−0.2
CL	L/h	14.7 (1.8)	14.6	14.0–15.2	−0.7
Q	L/h	5.81 (6.9)	5.73	3.83–7.64	−1.4
V3	L	33.8 (3.6)	33.6	30.5–36.5	−0.6
D1	h	0.281 (4.2)	0.278	0.211–0.309	−1.1
Food on D1	—	1.59 (3.8)	1.52	0.898–1.83	−4.4
CRCL on CL	—	0.460 (6.5)	0.486	0.417–0.570	5.7
Age on V2	—	0.286 (17.9)	0.260	0.114–0.372	−9.1
WT on V2	—	0.966 (13.5)	0.973	0.711–1.27	0.7

Parameter	Unit	Estimated mean (RSE%)	Shrinkage (%)	Bootstrap mean	95% CI	Bias (%)
Interindividual variability (ω2)						
η1	—	0.0515 (5.5)	14.7	0.0475	0.0289–0.0621	−7.8
η2	—	0.0635 (9.3)	29.6	0.0585	0.0359–0.0741	−7.9
η3	—	3.46 (7.8)	24.9	3.46	2.62–4.43	0.0
η4	—	0.0279 (8.7)	29.1	0.0284	0.0184–0.0394	1.8
Residual variability (σ2)						
ε1	—	0.0361 (1.5)	10.3	0.0362	0.0290–0.0429	0.3
ε2	—	2.35e-5 (6.5)	10.3	2.24e-5	8.20e-6–3.57e-5	−4.7

Abbreviations: 95% CI: 95% confidence interval; CL, clearance; D1, zero-order absorption rate; Ka, absorption rate constant; Q, intercompartmental clearance; RSE, standard error; Shrinkage, shrinkage value; TLAG, lag time in absorption; V2, central volume of distribution; V3, peripheral volume of distribution; ε1, proportional residual error; ε2, additive residuals; η1, interindividual variability of CL; η2, interindividual variability of V2; η3, interindividual variability of D1; η4, interindividual variability of IOV.

Notes: Deviation (%) = (Para bootstrap/Para final model-1) × 100%; Para represents the parameter estimate.

**FIGURE 1:**
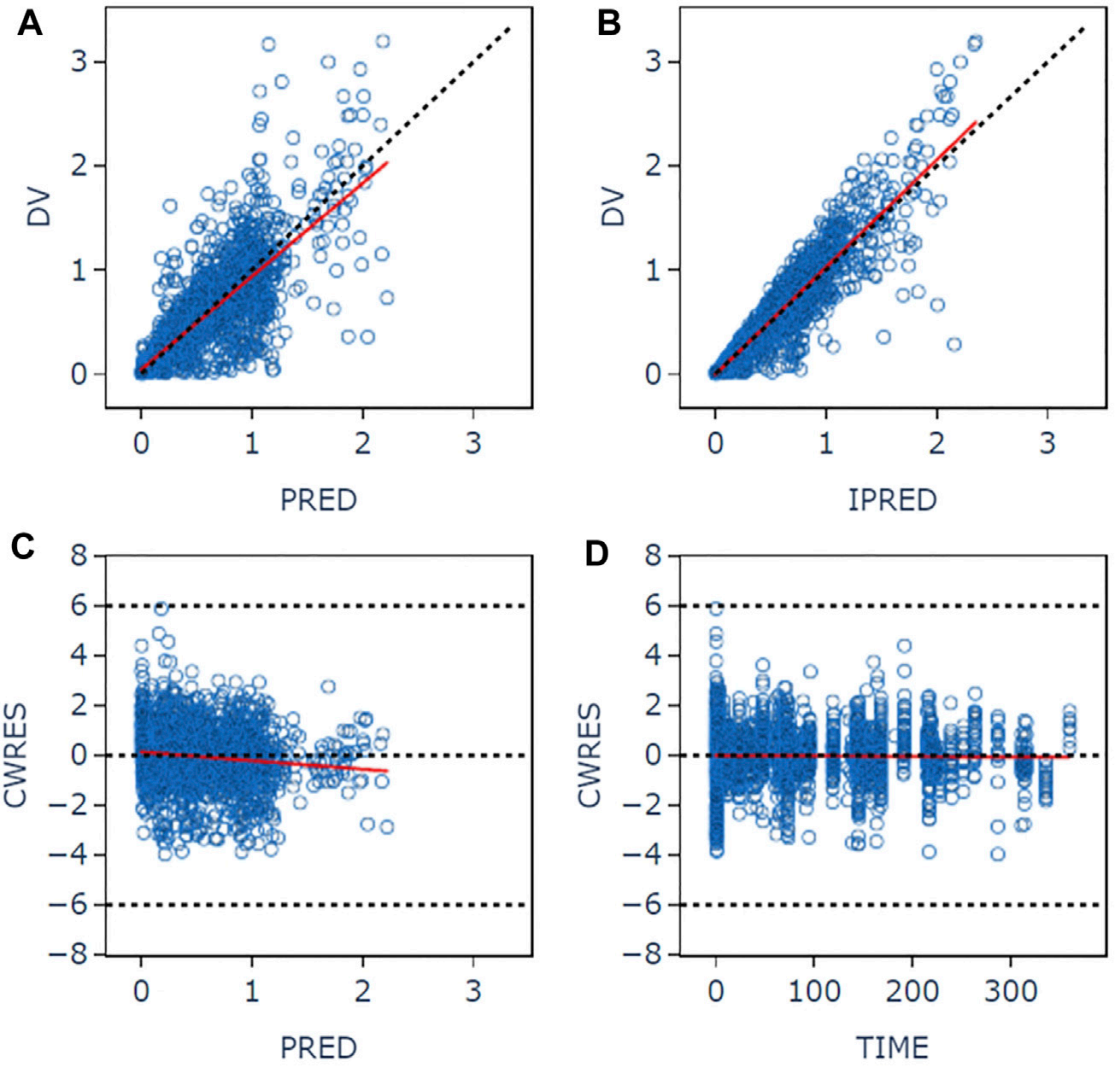
Goodness-of-fit plots for the final pharmacokinetic model. **(A)** Plot of observed concentrations versus population predictions. **(B)** Plot of observations versus individual predictions. **(C)** Conditional weighted residual versus population predictions. **(D)** Conditional weighted residual versus time. The open circles show the observations. The red line shows a smooth fit for the observations.

**FIGURE 2:**
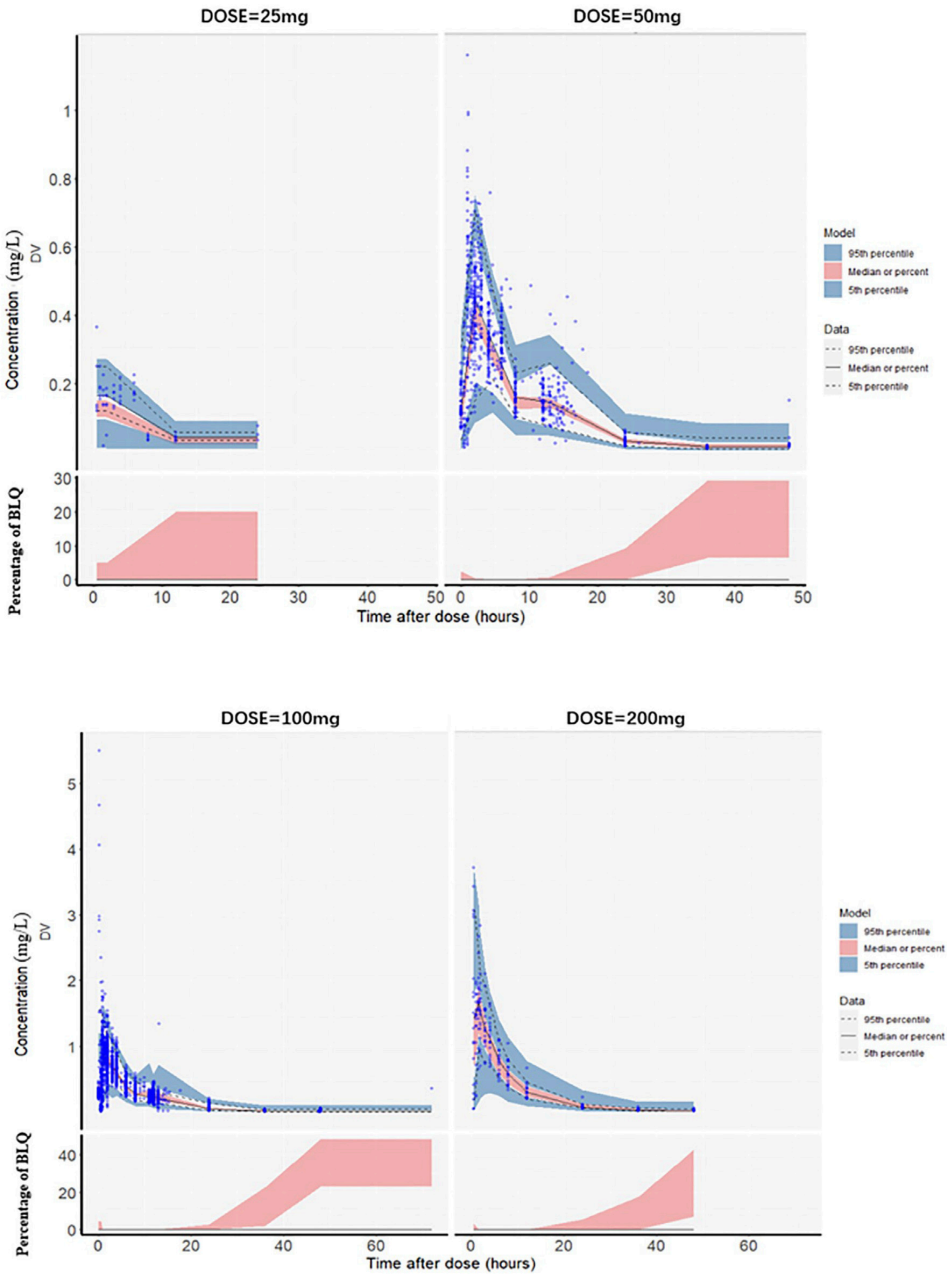
Visual predictive check plots for the plasma sitafloxacin concentration with different dosing regimens. The blue circles are the observed values, the black solid lines are the medians of the observed values, and the upper and lower black dotted lines correspond to the 95th and 5th quantiles, respectively. The red shaded area is the 95% confidence interval of the median predicted by the model, and the blue shaded area is the 95% confidence interval of the 5th and 95th quantile lines of the 1,000-time simulation data. The lower half of the chart: BLQ (Below the Limit of Quantitation) refers to the blood drug concentration being below the lower limit of quantification of the analytical method. The vertical axis represents the percentage of BLQ. The red shaded area indicates that as time extends, the probability of the blood drug concentration dropping below the limit of quantification gradually increases. (The quantitation limit of this study is 0.005–0.01 mg/L).

The goodness-of-fit plots for the final model are shown in Figure 1. The logarithm of population prediction (PRED), individual prediction (IPRED) and the observed plasma concentration (DV) of sitafloxacin fit well. There was no major bias for the conditional weighted residual (CERES) versus PRED or versus time. The medians of the parameter estimates from a 1000-bootstrap set analysis are shown in Table 2. The PopPK model was bootstrapped 1,000 times (945 successful bootstraps), and the convergence rate of the final covariate model operation was 94.5%. Each parameter estimate was within the range of the 95% confidence intervals, suggesting that the final model was robust. The visual predictive check plots (Figure 2) demonstrated that the majority of concentration points for all the subjects fell within the 95% confidence intervals predicted by the model, indicating that the model estimation results were stable.

### 2.3 Pharmacokinetics/Pharmacodynamics

**FIGURE 3:**
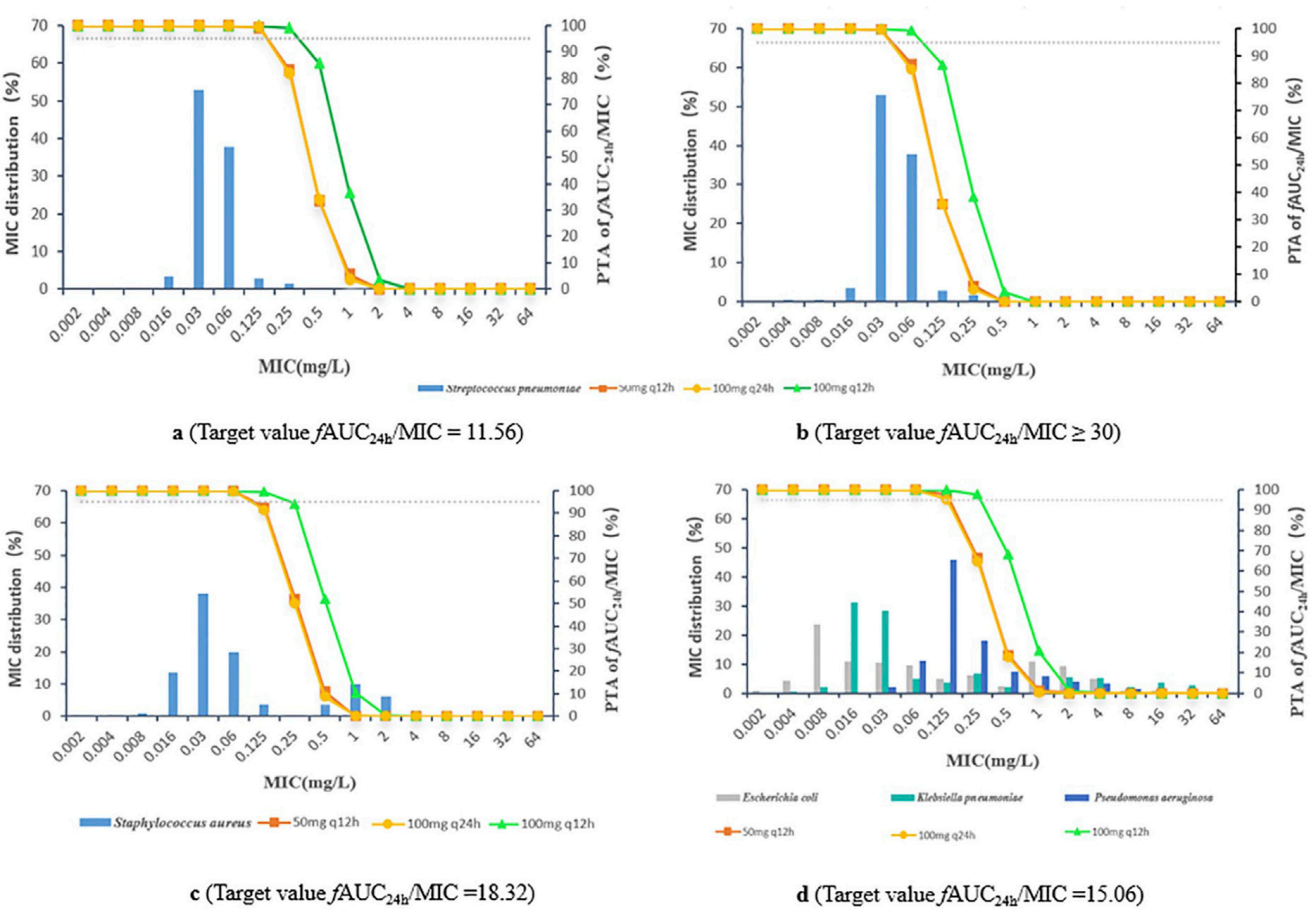
**(A)** *Streptococcus pneumoniae*, target value *f*AUC_24h_/MIC = 11.56 **(B)** *Streptococcus pneumoniae*, target value *f*AUC_24h_/MIC ≥30 **(C)** *Staphylococcus aureus*, target value *f*AUC_24h_/MIC = 18.32 **(D)** *Escherichia coli*, *Klebsiella pneumoniae*, and *Pseudomonas aeruginosa*, target value *f*AUC_24h_/MIC = 15.06.

For *Streptococcus pneumoniae*, the PTAs of *f*AUC_24h_/MIC for 50 mg q12 h, 100 mg q24 h and 100 mg q12 h were greater than 95% based on animal study targets when the MICs were ≤0.06, ≤0.06 and ≤0.125 mg/L, respectively. In terms of the clinical target, the three dosing regimens reached a PTA ≥95% for *Streptococcus pneumoniae,* with MICs ≤0.03, ≤0.03 and ≤0.06 mg/L, respectively. For *Staphylococcus aureus, Escherichia coli, Klebsiella pneumoniae,* and *Pseudomonas aeruginosa*, the PK/PD cut-off values were 0.06, 0.06 and 0.125 mg/L for the 50 mg q12 h, 100 mg q24 h and 100 mg q12 h dosing regimens, respectively (Figure 3; Table 3).

**TABLE 3:** PK/PD cut-offs and CFR for each bacterial strain under different sitafloxacin dosing regimens.

Dosing regimens for each PK/PD target	PK/PD cut-off (mg/L)	PTA (%)	CFR (%)
*Streptococcus pneumoniae*			
Animal infection model (*f*AUC_24h_/MIC = 11.56)			
50 mg q12 h	0.06	99.9	98.2
100 mg q24 h	0.06	99.9	98.2
100 mg q12 h	0.125	99.7	99.3
Clinical study (*f*AUC_24h_/MIC ≥30)			
50 mg q12 h	0.03	99.3	89.6
100 mg q24 h	0.03	99.1	89.2
100 mg q12 h	0.06	99.1	97.3
*Staphylococcus aureus*			
Animal infection model (*f*AUC_24h_/MIC = 18.32)			
50 mg q12 h	0.06	97.7	74.5
100 mg q24 h	0.06	97.9	74.6
100 mg q12 h	0.125	97	76.9
*Escherichia coli*
Animal infection model (*f*AUC_24h_/MIC = 15.06)			
50 mg q12 h	0.06	99.3	65.6
100 mg q24 h	0.06	99.1	65.5
100 mg q12 h	0.125	98.8	70.8
*Klebsiella pneumonia*			
Animal infection model (*f*AUC_24h_/MIC = 15.06)			
50 mg q12 h	0.06	99.3	71.8
100 mg q24 h	0.06	99.1	71.8
100 mg q12 h	0.125	98.8	76.6
*Pseudomonas aeruginosa*			
Animal infection model (*f*AUC_24h_/MIC = 15.06)			
50 mg q12 h	0.06	99.3	56.7
100 mg q24 h	0.06	99.1	56.3
100 mg q12 h	0.125	98.8	75.9

For *Streptococcus pneumoniae*, the CFRs were >90% at an *f*AUC_24h_/MIC of 11.56 for the 50 mg q12 h, 100 mg q24 h and 100 mg q12 h multidose dosing regimens; the CFRs of *f*AUC_24h_/MIC were approximately 89.2%–97.3% when *the f*AUC_24h_/MIC reached 30. The CFRs of all three dosing regimens for *Staphylococcus aureus, Escherichia coli, Klebsiella pneumoniae,* and *Pseudomonas aeruginosa* were 56.3%–76.9% (Table 3).

## 3 Discussion

In accordance with the recommended dosing regimen for sitafloxacin, adults are generally treated with 50 mg q12 h or 100 mg q24 h; for patients in which the drug exhibits unsatisfactory efficacy, the dosage may be increased to 100 mg q12 h. No dosage adjustment is required for patients with mild renal insufficiency (CRCL ≥ 50 mL/min). The purpose of this study was to establish a PopPK model based on PK data for sitafloxacin in healthy Japanese subjects and patients with target indications combined with phase I clinical PK data for sitafloxacin in healthy Chinese subjects and to accurately calculate the AUC_24h,ss_ of sitafloxacin at steady state in patients with normal renal function and mild renal insufficiency under different dosing regimens (50 mg q12h, 100 mg q24 h and 100 mg q12 h). In this study, PK/PD analysis was also performed to obtain PK/PD cut-off values for sitafloxacin against *Streptococcus pneumoniae, Staphylococcus aureus, Escherichia coli, Klebsiella pneumoniae,* and *Pseudomonas aeruginosa.*

The results from the final PopPK model revealed that the CRCL affected CL, WT and age affected V2, and food consumption status affected sitafloxacin absorption; these findings are similar to those reported in the literature. According to a previous study (Tanigawara et al., 2013), the PopPK base model for sitafloxacin was in agreement with a one-compartment model, but the two-compartment model more consistently fit the PK characteristics of sitafloxacin in this study. The bootstrap and VPC model validation results revealed that the constructed PopPK model was stable and reliable; therefore, this PopPK model can be further used in studies of the PK/PD of sitafloxacin.

A summary of the PK/PD cut-offs, ECOFFs, and MICs from clinical studies of sitafloxacin against target pathogens is provided in Table 4. When *f*AUC_24h_/MIC = 11.56 in the animal infection model was used as the target value, the PK/PD cut-off values (0.06∼0.125 mg/L) for sitafloxacin against *Streptococcus pneumoniae* under dosing regimenswere equal to or lower than the ECOFF (0.125 mg/L) and MIC_90_ (0.125 mg/L) values. However, these results are based on the more conservative PTA ≥95%, corresponding to the MIC as the PK/PD cut-off. If the PTA was set to be most often ≥90%, then the corresponding PK/PD cut-off values (0.125∼0.25 mg/L) can cover the other two values. When *f*AUC_24h_/MIC ≥30 in the clinical study was taken as the target value, the PK/PD cut-off values (0.03∼0.06 mg/L) were lower than the ECOFF and MIC_90_ values. Owing to the low MIC for *Streptococcus pneumoniae* isolated from patients enrolled in this clinical trial, the target *f*AUC_24h_/MIC may be overestimated, resulting in low PK/PD cut-offs. Therefore, on the basis of comprehensive consideration, the recommended PK/PD cut-off values for sitafloxacin against *Streptococcus pneumoniae* in patients were S ≤ 0.06 mg/L and R > 0.125 mg/L. The CFR of sitafloxacin against *Streptococcus pneumoniae* was >90%, suggesting that sitafloxacin is effective in treating infections caused by *Streptococcus pneumoniae*. The PK/PD cut-off values for sitafloxacin against the other four bacterial strain were 0.06∼ 0.125 mg/L. The PK/PD cut-off values for sitafloxacin against *Staphylococcus aureus, Escherichia coli* and *Klebsiella pneumoniae* were greater than or equal to the ECOFF value and not lower than the clinical MIC_50_ value (MIC_50_ ≤ 0.06 mg/L). However, for *Pseudomonas aeruginosa*, the PK/PD cut-off values were much lower than the ECOFF values and the clinical MIC values. Therefore, is sitafloxacin a poor treatment for infections caused by *Pseudomonas aeruginosa*? In a clinical trial evaluating the efficacy and safety of sitafloxacin in adult patients with community-acquired pneumonia, four patients with community-acquired pneumonia caused by *Pseudomonas aeruginosa* were successfully treated by oral administration of sitafloxacin at 100 mg qd or 100 mg q12h, and the MICs of sitafloxacin against isolated *Pseudomonas aeruginosa* ranged from ≤0.06–8^9^. This may be due to the good penetration of quinolones in the lung tissue site (ELF concentration/free drug blood concentration range 2–6) (Rodvold et al., 2011). The concentration of sitafloxacin in the lung tissue exceeds the MIC value, thereby achieving a bactericidal effect.

**TABLE 4:** Summary of PK/PD cut-offs, ECOFFs, and MICs from clinical studies of sitafloxacin against each bacterial strain.

Target value	*Streptococcus pneumoniae*		*Staphylococcus aureus*	*Escherichia coli*	*Klebsiella pneumoniae*	*Pseudomonas aeruginosa*
	Animal study target value *f*AUC_24h_/MIC = 11.56	Clinical target value *f*AUC_24h_/MIC ≥30	Target animal study value *f*AUC_24h_/MIC = 18.32	Target animal study value *f*AUC_24h_/MIC = 15.06	Target animal study value *f*AUC_24h_/MIC = 15.06	Target animal study value *f*AUC_24h_/MIC = 15.06
PK/PD cut-offs (mg/L)						
50 mg q12 h	0.06	0.03	0.06	0.06	0.06	0.06
100 mg q24 h	0.06	0.03	0.06	0.06	0.06	0.06
100 mg q12 h	0.125	0.06	0.125	0.125	0.125	0.125
ECOFFs (mg/L)	0.125		0.125	0.03	0.06	0.5
Clinical study data (mg/L)						
China (Li et al., 2021; Li et al., 2020)	9 strains		13 strains	82 strains	33 strains	7 strains
	MIC_50_ ≤ 0.06 MIC_90_ = 0.125		MIC_50_ ≤ 0.06 MIC_90_ ≤ 0.06	MIC_50_ ≤ 0.06 MIC_90_ = 1	MIC_50_ ≤ 0.06 MIC_90_ = 0.25	MIC_50_ = 0.5 MIC_90_ = 2
Japan (Tanigawara et al., 2013)	22 strains		11 strains	\	4 strains	4 strains
	MIC ≤0.025–0.39		MIC <0.025	\	MIC ≤0.025–0.78	MIC = 0.11–0.78

Owing to the small number of cases reported in the above clinical trials, the evaluation of the efficacy of sitafloxacin against infections caused by *Pseudomonas aeruginosa* needs to be supported by more clinical study data in the future.

The recommended dosage of sitafloxacin for patients with moderate renal insufficiency is 50 mg q24 h, as mentioned in the specifications for sitafloxacin. In this study, blood concentration data from 24 patients with moderate renal insufficiency were available, and we modelled the PK parameters of patients with moderate renal insufficiency and calculated the PK/PD boundaries at the recommended dosage. The results revealed that the PK/PD cut-offs for *Streptococcus pneumoniae, Staphylococcus aureus* and Gram-negative bacteria (*Escherichia coli, Klebsiella pneumoniae* and *Pseudomonas aeruginosa*) for the sitafloxacin dosing regimen of 50 mg q24 h were 0.06, 0.03, and 0.06 mg/L, respectively. The PK/PD cut-off for sitafloxacin against *Streptococcus pneumoniae* was 0.03 mg/L when the clinical target *f*AUC_24h_/MIC ≥ 30 was used. Considering the small number of patients with moderate renal insufficiency, this result is useful only for reference. As there were only 4 patients with severe renal insufficiency, PK simulation could not be performed, and the PK/PD cut-off was not obtained for this population.

## 4 Materials and methods

### 4.1 Data source

PK data from 12 clinical trials of sitafloxacin were combined in this study. Eleven of these clinical trials were performed in Japan and included studies on the PK profiles of sitafloxacin in healthy volunteers, elderly volunteers, patients with renal dysfunction and patients with respiratory tract infections. One clinical trial, a PK study on the oral administration of sitafloxacin in healthy Chinese subjects, was performed in China. In these clinical trials, the subjects received sitafloxacin as a single dose (3–200 mg) or multiple doses (50 or 100 mg q12 h or 100 mg q8h for 7–14 days). All the clinical trials were approved by the ethics committee of the corresponding study centre. All the subjects signed informed consent form before entry into the study. A detailed overview of the study design, treatment, population, and PK sampling for the 12 studies included in these analyses is presented in Supplementary File Table S1. The mean (SD) sitafloxacin plasma concentration-time profiles are illustrated in Supplementary File Figures S1–2.4.2. PopPK Model Development and Validation.

PopPK analysis was performed using nonlinear mixed-effects modelling via NONMEM 7.4 software (Icon Development Solutions, Ellicott City, MD, United States). The first-order conditional estimation method with interaction was applied to model development. The integrated management platform was powered by Mas Studio (version 1.6.2.1; Shanghai Bojia Medical Technology Co., Ltd.).

In the base model, the appropriate compartment model was selected, zero-order and first-order processes were attempted for absorption fitting, and elimination was performed via a first-order process. The final base model was selected by combining the objective function value (OFV), diagnostic plot and parameter stability.

The covariates that were screened included demographic information (age, weight, and sex), renal data (creatinine clearance and glomerular filtration rate), subject type (healthy volunteers vs patients), feeding status, test method, test institution and country (Japan and China). A covariate was retained in the forward inclusion model when the OFV decreased by 6.63 (P < 0.01) and in the backward elimination model when the OFV increased by more than 10.83 (P < 0.001).

The final model was evaluated by goodness-of-fit. Bootstrap analysis and a visual predictive check (VPC) were used to evaluate the robustness and predictive performance of the final model. The bootstrap analysis was repeated 1,000 times. For the VPC, 1,000 simulated replicates of the PK dataset were generated.

### 4.2 PK/PD analysis

The dosage of sitafloxacin for patients with normal renal function was 50 mg q12 h or 100 mg q24 h, and the dosage was increased to 100 mg q12 h if the efficacy was unsatisfactory. Moreover, no dosage or administration adjustment was required for sitafloxacin in patients with mild renal insufficiency (CRCL ≥ 50 mL/min). On the basis of the final PopPK model, the individual concentration–time profiles of patients with normal renal function and mild renal insufficiency (CRCL ≥ 50 mL/min) were simulated for sitafloxacin regimens of 50 mg q12 h, 100 mg q24 h, and 100 mg q12 h for 7 days. PK parameters such as the area under the steady-state concentration‒time curve (AUC_24h,ss_) were calculated according to the simulated PK profiles using NONMEM.

PD data were obtained from the MIC distribution frequency tables published on the EUCAST website (http://www.eucast.org). PK/PD analyses were performed in conjunction with *in vitro* PD data for sitafloxacin against *Streptococcus pneumoniae, Staphylococcus aureus, Escherichia coli, Klebsiella pneumoniae and Pseudomonas aeruginosa*.

In the mouse gastrocnemius muscle infection model, the *f*AUC_24h_/MIC targets for sitafloxacin against *Streptococcus pneumoniae*, *Staphylococcus aureus*, and Gram-negative organisms (*Escherichia coli*, *Klebsiella pneumoniae*, and *Pseudomonas aeruginosa*) were 11.56, 18.32, and 15.06, respectively. In a PK/PD study of Japanese patients with community-acquired respiratory infections, the *f*AUC_24h_/MIC target for sitafloxacin against *Streptococcus pneumoniae* was 30 (Tanigawara et al., 2013). Monte Carlo simulation was performed to calculate the PTA and CFR. When the PTA was greater than 95%, the highest value of the corresponding MIC range was defined as the PK/PD cut-off. The CFR, namely, the probability of the PK/PD index reaching the target against specific bacteria with various MIC levels, was used to evaluate the probability of the expected microbiological efficacy of the three regimens.

## 5 Conclusion

In conclusion, on the basis of national and international primary data, the PK/PD cut-offs for sitafloxacin against *Streptococcus pneumoniae, Staphylococcus aureus, Escherichia coli, Klebsiella pneumoniae, and Pseudomonas aeruginosa* in patients with normal renal function and mild renal insufficiency at dosages of 50 mg q12 h, 100 mg q24 h, and 100 mg q12 h, as determined by PopPK and Monte Carlo simulation, were S ≤ 0.06 mg/L and R > 0.125 mg/L, respectively. The PK/PD cut-off values need to be verified in clinical studies in the future. On the basis of the comparison of PK/PD cut-offs with ECOFFs, MICs in clinical studies and CFRs, sitafloxacin dosing regimens of 50 mg q12 h, 100 mg q24h and 100 mg q12 h were found to be effective in the treatment of *Streptococcus pneumoniae*-associated infections, but their efficacy against *Pseudomonas aeruginosa*-associated infections needs to be verified in clinical practice.

## Data Availability

The original contributions presented in the study are included in the article/Supplementary Material, further inquiries can be directed to the corresponding author.
