# Supplementary material for: Model-guided development of pharmacokinetic/pharmacodynamic cut-offs and evaluation of sitafloxacin dosing regimens against target pathogens
Source: Front Pharmacol. 2025 Feb 14;16:1476158. doi: 10.3389/fphar.2025.1476158 (PMC11868765; doi:10.3389/fphar.2025.1476158)
Supplement: Supplementary file 1 [file Table1.DOCX]

Table 1 Overview of information from clinical trials included in the PopPK analysis of sitafloxacin.

| **No.** | **Clinical Trial No.** | **Country** | **Dosing Regimen** | **Total Number of Subjects/Subjects Included in PK analysis** | **Subject Population** | **Blood Sample Collection** |
| --- | --- | --- | --- | --- | --- | --- |
| 1 | DU6859a-44 | Japan | 50 mg bid, 14 doses for 7 consecutive days | 115/109 | Patients with respiratory infection | Any time point |
|  |  |  | 100 mg bid, 14 doses for 7 consecutive days | 33/28 |  |  |
| 2 | DU6859a-19 | Japan | 50 mg, 1 dose | 6/5 | Patients with chronic respiratory infection | Before administration (0 h) and 1 h, 2 h, 4 h, 8 h, and 24 h after administration |
|  |  |  | 100 mg, 1 dose | 6/5 |  |  |
| 3 | DU6859a-37 | Japan | 50 mg, 1 dose | 12/12 | Subjects with insufficiency renal | Before administration (0 h) and 0.5 h, 1 h, 1.5 h, 2 h, 2.5 h, 3 h, 4 h, 6 h, 8 h, 12 h, 24 h, 36 h, and 48 h after administration |
| 4 | DU6859a-18 | Japan | 100 mg bid, 13 doses for 7 consecutive days | 29/29 | Healthy subjects | On Day 1, before administration (0 h) in the morning and 2 h, 4 h and 23 h after administration; on Day 2/Day 3/Day 5/Day 6, 23 h after administration in the morning; on Day 4, 2 h, 4 h and 23 h after administration in the morning; on Day 7, 2 h, 4 h, 24 h and 48 h after administration in the morning |
| 5 | DU6859a-23 | Japan | 100 mg bid, 27 doses for 14 consecutive days | 15/15 | Healthy subjects | On Day 1, before administration (0 h) in the morning and 2 h, 4 h and 23 h after administration; on Days 2-6/Days 8-13, 23 h after administration in the morning; on Day 7, 2 h, 4 h and 23 h after administration in the morning; on Day 14, 2 h, 4 h, 24 h and 48 h after administration in the morning |
| 6 | DU6859a-28 | Japan | 100 mg, 1 dose | 11/11 | Healthy subjects | Before administration (0 h) and 0.5 h, 1 h, 2 h, 3 h, 4 h, 6 h, 8 h, 12 h, 24 h and 48 h after administration |
| 7 | DU6859a-29 | Japan | 100 mg, 1 dose | 22/22 | Healthy subjects | Before administration (0 h) and 0.25 h, 0.5 h, 0.75 h, 1 h, 1.25 h, 1.5 h, 2 h, 4 h, 6 h, 8 h, 12 h and 24 h after administration |
| 8 | DU6859a-39 | Japan | 100 mg, 1 dose | 6/6 | Healthy subjects | Before administration (0 h) and 1 h, 2 h, 3 h, 4 h, 5 h, 6 h, 7 h, 8 h, 9 h and 10 h after administration |
| 9 | DU6859-01 | Japan | 3 mg, 1 dose | 2/2 | Healthy subjects | 3 mg or 10 mg, 1 dose: before administration (0 h) and 1 h, 2 h, 3 h, 4 h, 8 h, 12 h, 24 h after administration  25 mg or 50 mg or 100 mg or 200 mg, 1 dose: before administration (0 h) and 0.5 h, 1 h, 1.5 h, 2 h, 3 h, 4 h, 6 h, 8 h, 12 h, 24 h, 36 h, 48 h after administration  100 mg tid, 19 doses for 7 consecutive days: on Day 1, before administration (0 h) in the morning and 0.5 h, 1 h, 2 h, 3 h, 4 h, 6 h, 8 h, 12 h after administration; on Day 2/Day 3/Day 5/Day 6, before administration (0 h) in the morning; on Day 4, before administration (0 h) in the morning and 1 h, 2 h, 3 h, 4 h, 6 h, 8 h, 12 h after administration; on Day 7, before administration (0 h) in the morning and 1 h, 2 h, 3 h, 4 h, 6 h, 8 h, 12 h, 24 h, 48 h, 72 h after administration |
|  |  |  | 10 mg, 1 dose | 2/2 |  |  |
|  |  |  | 25 mg, 1 dose | 6/6 |  |  |
|  |  |  | 50 mg, 1 dose | 6/6 |  |  |
|  |  |  | 100 mg, 1 dose | 12/12 |  |  |
|  |  |  | 200 mg, 1 dose | 6/6 |  |  |
|  |  |  | 100 mg tid, 19 doses for 7 consecutive days | 6/6 |  |  |
| 10 | DU6859-02 | Japan | 50 mg bid, 13 doses for 7 consecutive days | 6/6 | Healthy subjects | On Day 1, before administration (0 h) in the morning and 0.5 h, 1 h, 1.5 h, 2 h, 3 h, 4 h, 6 h, 8 h, 12 h after administration; on Day 2/Day 3/Day 5/Day 6, before administration (0 h) in the morning; on Day 4, before administration (0 h) in the morning and 1 h, 2 h, 3 h, 4 h, 6 h, 8 h, 12 h after administration; on Day 7, before administration (0 h) in the morning and 1 h, 2 h, 3 h, 4 h, 6 h, 8 h, 24 h, 48 h after administration |
| 11 | DU6859a-05 | Japan | 100 mg bid, 13 doses for 7 consecutive days | 6/6 | Healthy subjects | On Day 1, before administration in the morning (0 h) and 0.5 h, 1 h, 1.5 h, 2 h, 3 h, 4 h, 6 h, 8 h, 12 h after administration on Day 2/Day 3/Day 5/Day 6, before administration in the morning (0 h) on Day 4, before administration in the morning (0 h) and 1 h, 2 h, 3 h, 4 h, 6 h, 8 h, 12 h after administration on Day 7, before administration in the morning (0 h) and 1 h, 2 h, 3 h, 4 h, 6 h, 8 h, 24 h, 48 h after administration |
| 12 | DU6859-A-A101 | China | 50 mg, 1 dose | 12/12 | Healthy subjects | Before administration (0 h) and 0.5 h, 1 h, 1.5 h, 2 h, 3 h, 4 h, 6 h, 8 h, 12 h, 24 h, 36 h, 48 h after administration |
|  |  |  | 100 mg, 1 dose | 12/12 |  | Before administration (0 h) and 0.5 h, 1 h, 1.5 h, 2 h, 3 h, 4 h, 6 h, 8 h, 12 h, 24 h, 36 h, 48 h after administration |
|  |  |  | 200 mg, 1 dose | 12/12 |  | Before administration (0 h) and 0.5 h, 1 h, 1.5 h, 2 h, 3 h, 4 h, 6 h, 8 h, 12 h, 24 h, 36 h, 48 h after administration |
|  |  |  | 100 mg bid, for 10 consecutive days (only 1 dose in the morning on Day 1, no dose on Day 2, bid from Day 3 to Day 9 (1 dose in the morning and 1 dose in the evening, with 12 hours interval), and only 1 dose in the morning on Day 10) | 12/12 |  | Blood samples were collected before administration (0 h) in the morning and 0.5 h, 1 h, 1.5 h, 2 h, 3 h, 4 h, 6 h, 8 h, 12 h, 24 h (Day 2/Day 10), 36 h (Day 2/Day 10) and 48 h (Day 3/Day 11) after administration; blood samples were collected to determine the trough concentration before administration (0 h) in the morning from Day 4 to Day 9 |


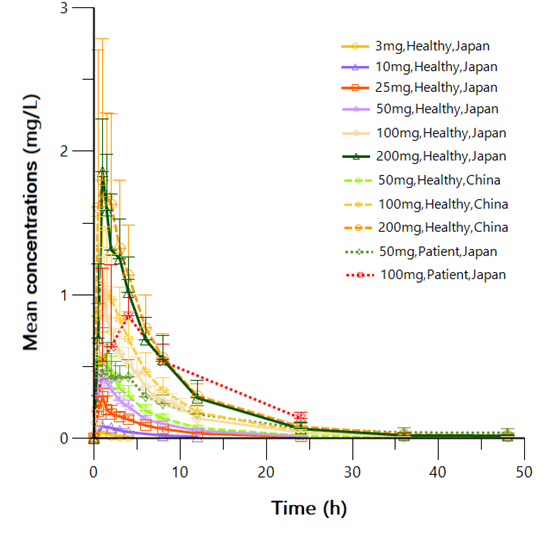
**(A)**

**(B)**


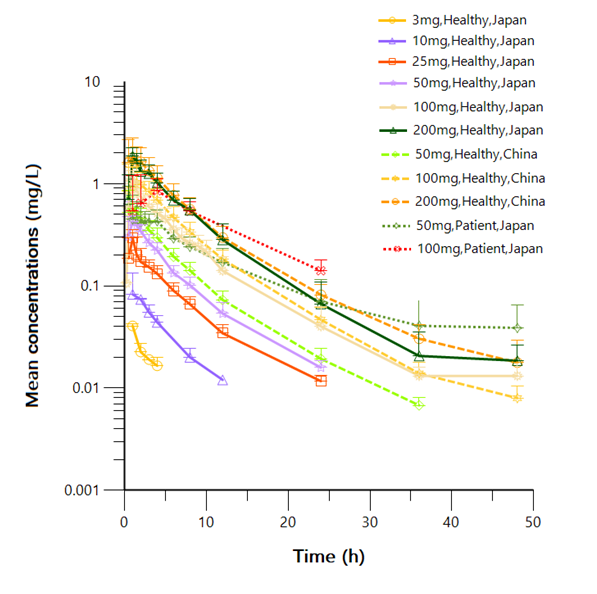


Figure1.Mean(±SD) sitafloxacin plasma concentration-time profiles after single-dose oral of 3 mg to 200mg in healthy subjects and patients in Japan and China. (A) The linear scale. (B) The semi-logarithmic scale. SD, standard deviation.


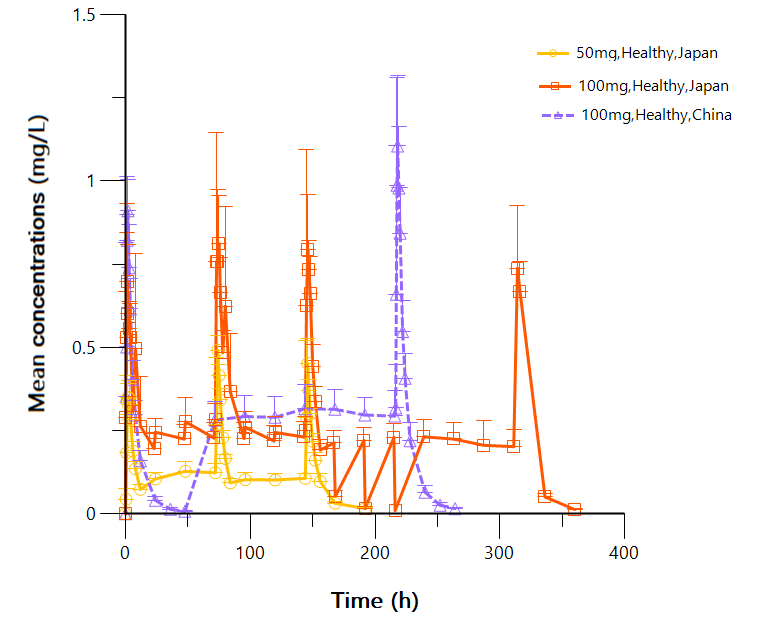
**(A)**


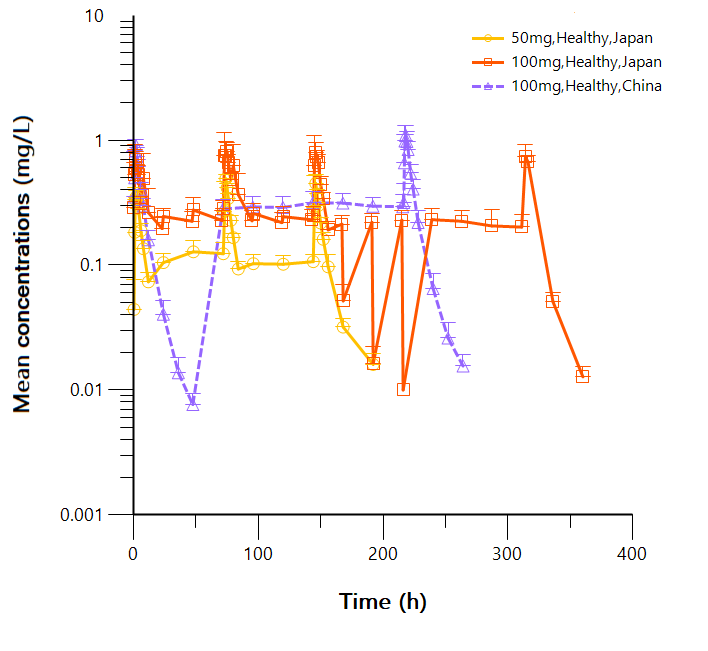


**(B)**

Figure2.Mean(±SD) sitafloxacin plasma concentration-time profiles after multi-dose oral of 50 mg and 100mg in healthy subjects in Japan and China. (A) The linear scale. (B) The semi-logarithmic scale. SD, standard deviation.
